# Chicoric acid: chemistry, distribution, and production

**DOI:** 10.3389/fchem.2013.00040

**Published:** 2013-12-31

**Authors:** Jungmin Lee, Carolyn F. Scagel

**Affiliations:** ^1^United States Department of Agriculture, Agricultural Research Service, Horticultural Crops Research Unit WorksiteParma, ID, USA; ^2^United States Department of Agriculture, Agricultural Research Service, Horticultural Crops Research UnitCorvallis, OR, USA

**Keywords:** phenolics, polyphenolics, cichoric acid, caffeic acid derivative, dicaffeoyltartaric acid, hydroxycinnamic acid, phenolic acid

## Abstract

Though chicoric acid was first identified in 1958, it was largely ignored until recent popular media coverage cited potential health beneficial properties from consuming food and dietary supplements containing this compound. To date, plants from at least 63 genera and species have been found to contain chicoric acid, and while the compound is used as a processing quality indicator, it may also have useful health benefits. This review of chicoric acid summarizes research findings and highlights gaps in research knowledge for investigators, industry stakeholders, and consumers alike. Additionally, chicoric acid identification, and quantification methods, biosynthesis, processing improvements to increase chicoric acid retention, and potential areas for future research are discussed.

## Introduction

Recent US consumer interest in boosting their dietary intake of chicoric acid followed popular-media coverage of claims that consumption of products containing chicoric acid had promising health benefits (Drazen, 2003). A recent literature search on chicoric acid reveals, not unsurprisingly, that a preponderance of the published research on chicoric acid is related to its potential medicinal uses (>50%) and research related to its chemistry (~18%), natural production in agriculture (~13%), and retention in foods (~18%) is lagging. Improved knowledge relating to chicoric acid biochemistry, how to enhance it in plant production, and how to retain its presence and activity in food and food products is needed.

**Figure 1:**
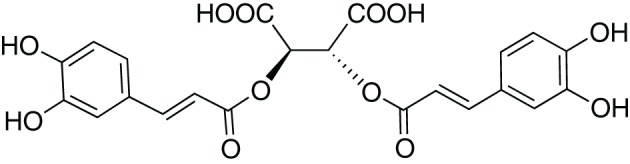
**Structure of L-chicoric acid**.

In 1958, while working on the leaves of chicory (*Cichorium intybus* L.) plants, Scarpati and Oriente (1958) isolated and identified a phenolic compound that was a tartaric acid ester of two caffeic acids (a hydroxycinnamic acid; Figure 1); they proposed naming it chicoric acid. Since that first discovery, chicoric acid has since been charted in many plant families, including those of seagrass, horsetail, fern, lettuce, and basil (Table 1). Other common names for chicoric acid are cichoric acid and dicaffeoyltartaric acid (Lee and Scagel, 2009), but for conciseness we will refer to this compound as chicoric acid.

**Table 1:** **Chicoric acid has been identified in the following plants**.

**Common name(s)**	**Order**	**Family**	**Genus and species**	**Plant parts evaluated and chicoric acid found in**	**References**
Burhead, Chapeu-de-couro	Alismatales	Alismatacease	*Echinodorus grandiflorus*	Leaves	Garcia Ede et al., 2010
Threelobe beggarticks, trifid burr marigold, marigold burr	Asterales	Asteraceae	*Bidens tripartita* L.	Aerial part	Pozharitskaya et al., 2010
Endive, cultivated endive, escarole	Asterales	Asteraceae	*Cichorium endivia* L.	Aerial part	Winter and Herrmann, 1986; Goupy et al., 1990; Degl'Innoocenti et al., 2008; Mascherpa et al., 2012
Chicory, radicchio	Asterales	Asteraceae	*Cichorium intybus* L.	Green/red and etiolated aerial part, roots, and seeds	Scarpati and Oriente, 1958, Bridle et al., 1984; Rees and Harborne, 1985; Winter and Herrmann, 1986; Chkhikvishvili and Kharebava, 2001; Mulinacci et al., 2001; Innocenti et al., 2005; Qu et al., 2005; Rossetto et al., 2005; Heimler et al., 2009; Jaiswal et al., 2011; Juskiewicz et al., 2011; Carazzone et al., 2013; Ritota et al., 2013; Ziamajidi et al., 2013
Smooth hawksbeard	Asterales	Asteraceae	*Crepis capillaris* L. Wallr.	Flower heads	Zidorn et al., 2005
Smooth purple coneflower	Asterales	Asteraceae	*Echinacea atroruebns* Nutt.	Aerial parts (flower heads, leaves, stems) and roots	Binns et al., 2002
Echinacea, black Samson Echinacea	Asterales	Asteraceae	*Echinacea augustifolia* DC.	Aerial part, roots, and pills (unknown parts)	Chkhikvishvili and Kharebava, 2001; Perry et al., 2001; Binns et al., 2002; Laasonen et al., 2002; Brown et al., 2010
Topeka purple coneflower	Asterales	Asteraceae	*Echinacea laevigata* (C.L. Boynt. and Beadle) S.F. Blake	Aerial parts (flower heads, leaves, stems) and roots	Binns et al., 2002; Pellati et al., 2005
Echinacea, pale purple coneflower, coneflower	Asterales	Asteraceae	*Echinacea pallida* Nutt.	Aerial parts and roots	Perry et al., 2001; Binns et al., 2002; Laasonen et al., 2002; Pellati et al., 2005; Brown et al., 2010; Sabra et al., 2012; Thomsen et al., 2012
Bush's purple coneflower	Asterales	Asteraceae	*Echinacea paradoxa* (J.B.S. Norton) Britton	Roots	Binns et al., 2002; Pellati et al., 2005
Echinacea, eastern purple coneflower, coneflower, purple coneflower	Asterales	Asteraceae	*Echinacea purpurea* L. Monech	Aerial parts (flower heads, leaves, stems) and roots	Wills and Stuart, 1999, 2000; Bergeron et al., 2000, 2002; Kim et al., 2000; Perry et al., 2001; Laasonen et al., 2002; Stuart and Wills, 2003; Pellati et al., 2005, 2011; Liu et al., 2006; Wu et al., 2007a; Araim et al., 2009; Lee and Scagel, 2009, 2010; Brown et al., 2010; Chen et al., 2010; Lee, 2010; Zhang et al., 2011; Sabra et al., 2012; Thomsen et al., 2012
Sanguin purple coneflower, purple coneflower	Asterales	Asteraceae	*Echinacea sanguinea* Nutt.	Aerial parts (flower heads, leaves, stems) and roots	Binns et al., 2002; Pellati et al., 2005
Wavyleaf purple coneflower	Asterales	Asteraceae	*Echinacea simulata* R.L. McGregor	Aerial parts (flower heads, leaves, stems) and roots	Binns et al., 2002; Pellati et al., 2005
Tennesseensis purple coneflower	Asterales	Asteraceae	*Echinacea tennesseensis* (Beadle) Small	Aerial parts (flower heads, leaves, stems) and roots	Binns et al., 2002; Pellati et al., 2005
Hairy cat's ear	Asterales	Asteraceae	*Hypochaeris radicata* L.	Flowering heads	Zidorn et al., 2005
Indian lettuce	Asterales	Asteraceae	*Lactuca indica* L.	Aerial parts	Kim et al., 2010
Iceberg lettuce, loose leaf lettuce, romaine lettuce, butterhead lettuce, baby lettuce	Asterales	Asteraceae	*Lactuca sativa* L.	Lettuce head (edible and inedible parts) and leaves	Winter and Herrmann, 1986; Tomas-Barberan et al., 1997; Gil et al., 1998; Cantos et al., 2001; Romani et al., 2002; Baur et al., 2004; Becker et al., 2004, 2013; Nicolle et al., 2004; Beltran et al., 2005; Kenny and O'Beirne, 2009; Oh et al., 2009; Chisari et al., 2010; Mulabagal et al., 2010; Jaiswal et al., 2011; Ribas-Agusti et al., 2011; Luna et al., 2012; Abu-Reidah et al., 2013; Mai and Glomb, 2013
Fall dandelion	Asterales	Asteraceae	*Leontodon autumnalis* L.	Flower head	Grass et al., 2006
Spiny sowthistle	Asterales	Asteraceae	*Sonchus asper* L. Hill	Leaves	Gatto et al., 2011
Common sowthistle, smooth sowthistle	Asterales	Asteraceae	*Sonchus oleraceus* L.	Leaves	Gatto et al., 2011; Ou et al., 2012
Common dandelion, dandelion	Asterales	Asteraceae	*Taraxacum officinale* F.H. Wigg.	Flowers, involucre bracts, leaves, stems, and roots	Williams et al., 1996; Chkhikvishvili and Kharebava, 2001; Schütz et al., 2005; Gatto et al., 2011; Park et al., 2011
Zucchini	Cucurbitales	Cucurbitaceae	*Cucurbita pepo* L.	Fruit	Iswaldi et al., 2013
Horsetail	Equisetales	Equisetaceae	*Equisetum* hybrids: *Equisetum arvense* × *palustre Equisetum* × *litorale Equisetum* × *rothmaleri Equisetum* × *torgesianum* act. non Rothm.	Part not specified	Veit et al., 1992
Field horsetail, common horsetail	Equisetales	Equisetaceae	*Equisetum arvense* L.	Sprouts (fertile) and gametophytes	Hasegawa and Taneyama, 1973; Veit et al., 1991, 1992; Hohlfeld et al., 1996
Scouringrush horsetail	Equisetales	Equisetaceae	*Equisetum hyemale* L.	Frond	Hasegawa and Taneyama, 1973
Causasian whortleberry	Ericales	Ericaceae	*Vaccinium arctostaphylos* L.	Leaves, fruits	Chkhikvishvili and Kharebava, 2001
Peanut	Fabales	Fabaceae	*Arachis hypogaea* L.	Leaf terminals	Snook et al., 1994
Snakeroot, serpentine roots, chandra	Gentianales	Apocyanaceae	*Rauvolfia serpentine* (L.) Benth. Ex Kurz.	Shoots and roots.	Nair et al., 2013
Sickle seagrass, thalassia	Hydrocharitales	Hydrocharitaceae	*Thalassia hemprichii* Asch.	Leaves	Qi et al., 2012
Borage, starflower, common borage	Lamiales	Boraginaceae	*Borago officinalis* L.	Leaves	Gatto et al., 2011
Basil, sweet basil	Lamiales	Lamiaceae	*Ocimum basilicum* L.	Aerial parts and roots	Lee and Scagel, 2009, 2010; Shiga et al., 2009; Lee, 2010; Nguyen et al., 2010; Kwee and Niemeyer, 2011; Scagel and Lee, 2012
African basil, efinrin	Lamiales	Lamiaceae	*Ocimum gratissimum* L.	Leaves	Ola et al., 2009
Cat's whiskers, orthoshiphon	Lamiales	Lamiaceae	*Orthosiphon stamineus* Benth.	Leaves	Olah et al., 2003
Rabdosia	Lamiales	Lamiaceae	*Rabdosia rubescens*	Leaves	Tang et al., 2011
Mules foot fern	Marattiales	Marattiaceae	*Angiopteris lygodiifolia*	Frond	Hasegawa and Taneyama, 1973
Lesser neptune grass	Najadales	Cymodoceaceae	*Cymodocea nodosa* (Ucria) Asch.	Leaves (fresh and detrital) and rhizomes	Grignon-Dubois and Rezzonico, 2013
Manatee grass	Najadales	Cymodoceaceae	*Syringodium filiforme* Kutz.	Leaves	Nuissier et al., 2010
Mediterranean tapeweed, neptune grass	Najadales	Posidoniaceae	*Posidonia oceanica* L. Delile	Shoots, leaves, and roots	Cariello and Zanetti, 1979; Cariello et al., 1979; Agostini et al., 1998; Dumay et al., 2004; Haznedaroglu and Zeybek, 2007; Heglmeier and Zidorn, 2010
Eelgrass, seawrack	Najadales	Zosteraceae	*Zostera marina* L.	Leaves	Pilavtepe et al., 2012
Japanese deer fern	Polypodiales	Blechnaceae	*Struthiopteris niponica, Blechnum nipponicum*	Frond	Hasegawa and Taneyama, 1973
Tree fern	Polypodiales	Cyatheaceae	*Cyathea fauriei*	Frond	Hasegawa and Taneyama, 1973
Squirrel's foot fern	Polypodiales	Davalliaceae	*Davallia mariesii*	Frond	Hasegawa and Taneyama, 1973
Western brackenfern	Polypodiales	Dennstaedtiaceae	*Pteridium aquilinum* (L.) Kuhn	Frond	Hasegawa and Taneyama, 1973
Japanese painted fern	Polypodiales	Dryopteridaceae	*Athyrium niponicum*	Frond	Hasegawa and Taneyama, 1973
Bladder fern	Polypodiales	Dryopteridaceae	*Cystopteris japonica*	Frond	Hasegawa and Taneyama, 1973
Wood fern	Polypodiales	Dryopteridaceae	*Dryopteris africana*	Frond	Hasegawa and Taneyama, 1973
Champion's wood fern	Polypodiales	Dryopteridaceae	*Dryopteris championii*	Frond	Hasegawa and Taneyama, 1973
Autumn fern, Japanese wood fern, Copper shield fern	Polypodiales	Dryopteridaceae	*Dryopteris erythrosora*	Frond	Hasegawa and Taneyama, 1973
Sensitive fern	Polypodiales	Dryopteridaceae	*Onoclea sensibilis* L.	Frond	Hasegawa and Taneyama, 1973
Trifid holly fern	Polypodiales	Dryopteridaceae	*Polystichum tripteron, Asipdium tripteron, Dryopteris triptera*	Frond	Hasegawa and Taneyama, 1973
Manchurian fern	Polypodiales	Dryopteridaceae	*Woodsia manchuriensis*	Frond	Hasegawa and Taneyama, 1973
Forked fern	Polypodiales	Gleicheniaceae	*Gleichenia japonica*	Frond	Hasegawa and Taneyama, 1973
Japanese climbing fern	Polypodiales	Lygodiaceae	*Lygodium japonicum* (Thunb.) Sw.	Frond	Hasegawa and Taneyama, 1973
Royal ferm	Polypodiales	Osmundaceae	*Osmunda regalis* L.	Frond	Hasegawa and Taneyama, 1973
Polypody	Polypodiales	Polypodiaceae	*Neocheiropteris ensata, Polypodium ensatum, Neolepisorus ensatus,* and numerous other synonyms.	Frond	Hasegawa and Taneyama, 1973
Maidenhair fern	Polypodiales	Pteridaceae	*Adiantum monochlamys*	Frond	Hasegawa and Taneyama, 1973
Carrot fern, Japanese claw fern, claw fern	Polypodiales	Pteridaceae	*Onychium japonicum*	Frond	Hasegawa and Taneyama, 1973
Cretan brake	Polypodiales	Pteridaceae	*Pteris cretica* L.	Frond	Hasegawa and Taneyama, 1973
Chinese firethorn	Rosales	Rosaceae	*Pyracantha fortuneana* Li	Fruit	Zhao et al., 2013

They are alphabetically listed by order, family, genus, and species (USDA-NRCS, 2013; and reference listed in table). Common names and the plant fractions used in the referenced research papers are also summarized. Multiple genus and species listed within a cell are hybrids or synonyms.

Reports have indicated that chicoric acid helps a plant protect itself from insects and infection from viruses, bacteria, fungi, and nematodes (Cariello and Zanetti, 1979; Rees and Harborne, 1985; Snook et al., 1994; Nishimura and Satoh, 2006); and that it aids in wound healing in plants after mechanical damage (Tomas-Barberan et al., 1997). Beyond the better understood roles it has in seed germination, additional research is needed to better understand why plants produce chicoric acid and to corroborate theories that phenolic acids defend against microbial and herbivore attack by acting as deterrents, toxins, or signaling molecules (Harborne, 1979; Gallagher et al., 2010; Mandal et al., 2010). General summaries of phenolics' roles in ecosystem (Hattenschwiler and Vitousek, 2000) and ecophysiology (Cheynier et al., 2013) are available.

The significance of plant phenolics to humans is still developing. While individual phenolics have been used in plant systematics and have become recognized for their utility in quality monitoring of crops and product manufacturing, they are now being investigated for possible human health benefits. A thorough identification of phenolics in foods will aid research in determining food and dietary supplement authenticity, quality assurance standards, and health investigations (Winter and Herrmann, 1986; Stuart and Wills, 2003; Lee, 2010, 2014; Lee and Scagel, 2009, 2010; Sanzini et al., 2011). With our current awareness in eating a healthy diverse diet, and the recent attention chicoric acid has received, we consider this review necessary to summarize the present data on plant sources of chicoric acid and factors that may influence their identification, production, and use.

Our objective was to compile scientific findings on chicoric acid to aid future work: including its recognition, its known prevalence within the plant kingdom, its distribution throughout a plant, growing condition effects upon it, and processing techniques to retain its quality within final products.

## Identification, synthesis, and biosynthesis chicoric acid

### Obtainability, synthesis, and forms

Chicoric acid can be obtained from isolated and purified plant materials (Table 1) or synthesized (Scarpati and Oriente, 1958; Synoradzki et al., 2005). Pure chicoric acid is a white powder (Synoradzki et al., 2005; personal observation), and now available as a purified standard via numerous chemical companies (e.g., Cerilliant Chemical Company, Indofine Chemical Company, Inc., Sigma-Aldrich Co, LLC, etc.). The most abundant natural form is L-chicoric acid [i.e., (-)-chicoric acid, 2,3-dicaffeoyl-L-tartaric acid, 2,3-*O*-dicaffeoyltartaric acid, 2R,3R-*O*-dicaffeoyltartaric acid, or di-*E*-caffeoyl-(2R-3R)-(-)-tartaric acid] and has been reported in the majority of the plants listed in Table 1, but the stereoisomer *meso*-chicoric acid (i.e., dicaffeoyl-*meso*-tartaric acid or di-*E*-caffeoyl-(2R-3S)-(-)-tartaric acid) has also been reported at a lesser levels in horsetail sprouts (*Equisetum arvense* L.) (Veit et al., 1991, 1992; Hohlfeld et al., 1996), iceberg lettuce (*Lactuca sativa* L.) (Baur et al., 2004; Luna et al., 2012), and purple coneflower (*Echinacea purpurea* L. Monech) (Perry et al., 2001).

Identification of L-chicoric acid and *meso*-chicoric acid in plant samples has not been consistent. L-chicoric acid and an isomer (unknown form) were reported in chicory leaves (Heimler et al., 2009). Both L-chicoric acid and *meso*-chicoric acid were found in iceberg lettuce (Baur et al., 2004; Luna et al., 2012). *Equisetum arvense* L. fertile sprouts and gametophytes contained *meso*-chicoric acid and no L-chicoric acid (Veit et al., 1992), but Hasegawa and Taneyama (1973) reported only L-chicoric acid in *E. arvense* L. (unspecified growth stage of tissue). Hasegawa and Taneyama (1973) found L-chicoric acid in numerous fern frond samples (Table 1), though Veit et al. (1992) reported no L-chicoric acid and only *meso*-chicoric acid in their fern samples.

In some cases the *meso*-chicoric acid identified from plant extracts may have been due to isomerization of L-chicoric acid after sample extraction and/or purification (Snook et al., 1994; Perry et al., 2001). Snook et al. (1994) showed that a small amount of L-chicoric acid in peanut leaf terminal methanol extract isomerized into *meso*-chicoric acid at room temperature. Although three accounts from a single research group confirmed by Nuclear Magnetic Resonance (NMR) the tartaric acid structure of purified *meso*-chicoric acid of horsetail barren sprouts (Veit et al., 1991, 1992; Hohlfeld et al., 1996), an independent report found L-chicoric acid, but no *meso*-chicoric acid in horsetail (Hasegawa and Taneyama, 1973).

Differences in chicoric acid forms among research reports may be a result of the methods used for sample extraction and identification. L-chicoric acid and its isomers can be distinguished by 2D-paper chromatography. However, separation via High Performance Liquid Chromatography (HPLC), L- and D-chicoric acids (if D-chicoric acid exists) co-elute (Williams et al., 1996; Baur et al., 2004). In some cases, *meso*-chicoric acid was found to elute immediately after L-chicoric acid (Snook et al., 1994; Baur et al., 2004) in reversed-phase HPLC conditions. Thin Layer Chromatography (TLC) separates L-chicoric acid from *meso*-chicoric acid (Veit et al., 1991, 1992). Phenolic acid conjugated to *meso-*tartaric acid is rarely reported, but *p*-coumaroyl-*meso*-tartaric acid has been found in spinach (Winter and Herrmann, 1986; Bergman et al., 2001), indicating *meso*-tartaric acid conjugation to phenolic acids can occur naturally, however, it has yet to be confirmed that it formed before extraction. Work is still needed to confirm if *meso*-chicoric acid is present naturally.

### Identification and quantification

Chicoric acid identification and quantification can be achieved by either Planar (Paper or Thin Layer) Chromatography (Scarpati and Oriente, 1958; Rees and Harborne, 1985; Nicolle et al., 2004), HPLC coupled with a Diode Array Detector (DAD) and/or Mass Spectrometer (MS) Detector, or NMR spectroscopy (Lee and Scagel, 2009; Nuissier et al., 2010; Juskiewicz et al., 2011; Ritota et al., 2013).

Chicoric acid spectra by NMR can be found in Nuissier et al. (2010). Infrared spectra of chicoric acid can be found in Scarpati and Oriente (1958). If a purified chicoric acid standard is unavailable, than an extract of *E. purpurea*, chicory, or dandelion (since it is their main phenolic) can be made to aid peak identification (Williams et al., 1996; Lee and Scagel, 2009, 2010; Juskiewicz et al., 2011; and additional references listed in Table 1).

A clear UV-visible spectra (maximum absorption at 330 with a 300 nm shoulder) (Scarpati and Oriente, 1958; Pellati et al., 2005; Lee and Scagel, 2009) and a HPLC chromatogram of chicoric acid, and other caffeic acid derivatives, elution is available in past works (Bergeron et al., 2000; Kim et al., 2000; Baur et al., 2004; Nicolle et al., 2004; Lee and Scagel, 2009). A HPLC method (INA-Institute for Nutraceutical Advancement method 106.000) from NSF (National Science Foundation) International (Ann Arbor, MI, USA) is accessible as well. Clear chicoric acid mass spectra examples can be found in Shiga et al. (2009) and Mulinacci et al. (2001); mother and fragmented masses have been reported in numerous papers with various MS settings, so one of these references can be used for comparison with a comparable analysis (Mulinacci et al., 2001; Baur et al., 2004; Lee and Scagel, 2009; Shiga et al., 2009; Pellati et al., 2011; Ribas-Agusti et al., 2011; Carazzone et al., 2013).

Capillary electrophoresis (with DAD) has also been evaluated in chicoric acid quantification in *E. purpurea* dried press juice (Manèek and Kreft, 2005). The ability to predict chicoric acid production in *E. purpurea* plants by an extrapolation model using DNA fingerprinting and HPLC concentration results has also been explored (Baum et al., 2001). Developing rapid and accurate techniques for chicoric acid identification and quantification as well new methods for authenticating and predicting production in plants will aid future work by researchers, agronomists, and manufacturers.

### Extraction

Sample preparation has often been overlooked in quality analysis research, even though as the first laboratory stage of chemical analysis it has great consequences for the results (Williams et al., 1996; Bergeron et al., 2000; Stuart and Wills, 2000; Perry et al., 2001; Lee and Scagel, 2009; Kim and Verpoorte, 2010; Lee et al., 2012). Sample preparation can impact not only accurate identification of chicoric acid (e.g., L- vs. *meso-* form of chicoric acid) but also quantitative analyses. For example, Perry et al. (2001) showed >50% loss of chicoric acid when water was substituted for ethanol as the extraction solvent. Sample harvest timing (growth stage/development), handling, preparation, hydrolysis, and purification steps for optimum phenolic retention are areas to carefully consider when undertaking a detailed analysis.

We (Lee and Scagel, 2009, 2010; Lee, 2010; Scagel and Lee, 2012) were the first to discover that the second principal phenolic in basil (*Ocimum basilicum* L.) leaves was chicoric acid. Our suspicion is that identification of this basil compound was overlooked for so long was due to chicoric acid's prompt and rapid degradation during extraction procedures (Perry et al., 2001; Lee and Scagel, 2009). Lack of commercially accessible standard, in the past, also contributed to the delay in identification in basil (Lee and Scagel, 2009). In addition to extraction and analysis technical differences, a variety of sample preparation conditions contributed toward the disparity of reported chicoric acid levels in the literature. We demonstrated that blanching was a straightforward initial sample extraction step critical for high retention of phenolic compounds in samples high in native enzymes (Lee et al., 2002; Lee and Scagel, 2009; Zhang et al., 2011). Optimal extraction procedures for chicoric acid may differ from other compounds that may be of interest in plant samples. Chicoric acid extraction performance differed from alkamide extraction in *E. purpurea* roots and shoots (Stuart and Wills, 2000). This work also demonstrated other critical considerations for optimizing extraction procedures including the influence of particle size (smaller particles of root material permitted greater chicoric acid extraction), solid/solute to extraction solvent ratio (1 part solid to 8 parts solvent), extraction temperature (60°C), and ethanol solvent to water ratio (60% ethanol: 40% water). The ideal extraction condition for maximum alkamide was different than that for chicoric acid (90% ethanol: 10% water at 20°C; Stuart and Wills, 2000).

Different solvent systems for extraction have been evaluated and efficiency of extraction can vary between phenolics because these compounds can vary in their polarity and accessibility. Extraction of chicoric and chlorogenic acids in *Rauvolfia serpentina* (L.) Benth. Ex Kurz shoots and roots was greatest in aqueous acetonitrile > acidified (HCl) acetonitrile > aqueous methanol > aqueous ethanol > acidified (HCl) methanol > acidified (HCl) ethanol > 60°C water > ambient temperature water (Nair et al., 2013). In contrast, acidified (HCl) acetonitrile was considered a more superior extracting solution for caftaric and caffeic acids, and aqueous methanol for quercetin-rutinoside (a flavonol). Chicoric acid in *E. purpurea* samples was found to degrade or conjugate under conditions that inhibit degradation of caffeic acid derivatives (Nüsslein et al., 2000).

Current routine sample extraction procedures for chicoric acid are time consuming and labor intensive, and there has been little research on optimizing extraction for specific plants, plant structures, and multiple target compounds. Sample processing procedures in the future that allow for rapid sampling of a large number of samples for chicoric acid analyses are needed improve the quality and reliability of research results from greenhouse and field experiments.

### Biosynthesis

The biosynthetic pathway of L-chicoric acid (the most abundant form) is still not well known; although it is generally understood to form via the shikimic acid/phenylpropanoid pathway as other phenolic acids, analogous to the conjugation of caffeic acid derivatives of rosmarinic acid or chlorogenic acid (Kuhnl et al., 1987; Shetty, 2001; Petersen and Simmonds, 2003; Petersen et al., 2009). Overviews of phenolic biosynthetic pathway were well reviewed by Dixon and Paiva (1995); Vogt (2010), and Cheynier et al. (2013). To date, we found no published research on the specific enzymes involved in L-chicoric acid biosynthesis. While it is possible that some *meso*-chicoric acid findings may have occurred from isomerization of L-chicoric acid in methanol extracts prior to compound separation by HPLC, there have been some efforts in identifying the enzymes (e.g., hydroxycinnamoyltransferases) involved in *meso*-chicoric acid biosynthesis (Hohlfeld et al., 1996). Techniques using hairy root cultures (Liu et al., 2012) and molecular analyses (Baum et al., 2001; Rana and Chandra, 2006) may help expand our knowledge of chicoric acid biosynthesis in the future.

## Plant kingdom and within plant distribution

### Variation among plant taxa

To date, chicoric acid has been found in plants of at least 13 orders, 25 families, and 63 genera and species (listed in Table 1). It has been most often reported in either the family Asteraceae (Aster family)—20 genera and species, or the family Dryopteridaceae (Wood fern family)—8 genera and species. Production of chicoric acid does not appear to be ubiquitous in taxon within a plant family or sub-family. Chicoric acid has been detected in leaves of many genera in the Asteraceae, however, some genera within this family may not produce chicoric acid in leaves (e.g., *Achillea*, *Arnica*, *Cnicus*, *Echinops*, *Inula*, *Petasites*, *Solidago*, and *Tanacetum*; Jaiswal et al., 2011). Two genera within the Cichorioideae and Asteroideae sub-families of the Asteraceae are well-known for their chicoric acid production (e.g., *Cichorium*, *Echinacea*) yet chicoric acid has not been detected in other genera within these plant sub-families (8 genera listed above; Jaiswal et al., 2011).

Chicoric acid production varies within genera and within species. Within the genus *Echinacea* there is a wide range of chicoric acid production (Perry et al., 2001; Binns et al., 2002; Pellati et al., 2005; Sabra et al., 2012), with *E. purpurea* containing the greatest concentration of the nine species (Table 1). So, it is not surprising that among three popular and widespread *Echinacea* dietary supplements, preparations of *E. purpurea* contained higher levels of chicoric acid than either *E. pallida* or *E. augustifolia* (Perry et al., 2001; Binns et al., 2002; Pellati et al., 2005). Chicoric acid can also vary greatly between cultivars; Kwee and Niemeyer (2011) examined 15 basil cultivars and reported a chicoric acid range from 3 to 278 mg 100 g^−1^ dry weight (93 fold difference; and Ribas-Agusti et al. (2011) recorded chicoric acid concentrations from 23 to 1388 mg 100 g^−1^ fresh weight when they evaluated 13 cultivars of romaine lettuce.

### Variation among plant structures

There is some confusion surrounding what part of the plant is suitable for use in herbal medicine particularly in the US. The European Medicine Agency (London, England) maintains a list of the plant fractions approved for human herbal usage. For example, the European Medicine Agency lists the roots of *E. augustifolia* DC. and *E. pallida* Nutt. as recognized for herbal medicine, and the whole plant (aerial and roots) of *E. purpurea* based on traditional uses and scientific data. Chicoric acid can vary widely with plant age and portion. For example, in roots and shoots of *E. purpurea* grown ranged from 203 to 3855 mg 100 g^−1^ dry weight, an over 18 fold difference in both young and mature plant fractions (Qu et al., 2005).

Phenolic distribution within a plant are known to vary. We (Lee and Scagel, 2010) reported chicoric acid allocation of flowering *E. purpurea*, measuring over a 4 fold difference from sections highest to those lowest: leaves > roots > flowers > stems; 93–391 mg 100 g^−1^ fresh weight of fraction. Similar findings were reported by Molgaard et al. (2003) where chicoric acid in *E. purpurea* leaves > rootstock > flower head ≈ root > stem (4.5 fold difference; 930–4240 mg 100 g^−1^ dry weight). In contrast, chicoric in different plant parts of *E. purpurea* were reported in the descending order: flowers > leaves > stems > roots (compared in dry weight; Lin et al., 2011). Similar discrepancies among plant structures in chicoric acid accumulation were reported for Neptune grass (*Posidonia oceanica* L. Delile) where no chicoric acid was detected in leaves (Dumay et al., 2004) but others found it in leaves (Cariello and Zanetti, 1979; Heglmeier and Zidorn, 2010). Conflicting reports of chicoric acid accumulation among plant structures have also been reported for *E. angustifolia* where chicoric acid has (Hu and Kitts, 2000; Zheng et al., 2006) and has not (Li and Wardle, 2001) been detected in roots, and has (Zheng et al., 2006) and has not (Sloley et al., 2001) been detected in leaves. Differences in plant age and growing environment among these studies may account for the differences in how much chicoric acid accumulated in different structures.

Plant development and growing condition may influence accumulation of chicoric acid within a plant structure. For example, green leafy parts of endives were found higher in chicoric acid than the etiolated leaves, and endive green veins were slightly higher than white veins (Goupy et al., 1990). Outer leaves from lettuce (numerous cultivars and genotypes evaluated) were higher in chicoric acid than inner leaves (Winter and Herrmann, 1986; Hohl et al., 2001). Chicoric acid concentration coincided with color within a loose-leaf lettuce head, red > green > white, which matched from outer leaves, to inner leaves, and to midribs (Gil et al., 1998).

An uneven distribution of phenolics within plants is not surprising, as portions' susceptibility to biotic stresses naturally (e.g., defense or reproduction), results in supply dependent upon the plant's need (Snook et al., 1994; Dixon and Paiva, 1995; Harborne, 1999). Understanding what factors regulate expected chicoric acid allocation in plants could be useful to tailor the concentration of commercial products by including (or not including) certain plant fractions.

### Impact on consumer products

Supplements and other consumer products have shown even greater variation in chicoric acid content than reported for plant samples. For example, Molgaard et al. (2003) measured a 389 fold difference (from not detectable to 389 mg 100 mL^−1^; *n* = 13) of chicoric acid in *Echinacea* extracts, and a 3460 fold difference (from not detectable to 3460 mg 100 g^−1^; *n* = 6) of chicoric acid in *Echinacea* capsules. A range of 410–2140 mg 100 g^−1^ dry weight was reported for *E. purpurea* root and aerial samples (*n* = 62) purchased from herb traders (Wills and Stuart, 1999), and a range of 310–877 mg 100 g^−1^ dry weight (*n* = 24 accessions) in fall dandelion flower heads (*Leontodon autumnalis* L.; Grass et al., 2006). The diverse chicoric acid concentrations found within the basil, lettuce, and *Echinacea* samples summarized above emphasize the caution necessary when investigating chicoric acid's possible health benefits, especially if the evaluation is from a single form/sample of these plant materials.

Conflicting reports of chicoric acid accumulation in certain plant taxon can impact the potential for certain products to provide consumers with chicoric acid. Some of the chicoric acid reports listed in Table 1 have yet to be independently confirmed (e.g., peanut shoots, zucchini, or Chinese firethorn fruit). For example, Snook et al. (1994) reported chicoric acid concentration in nine varieties of peanut (*Arachis hypogaea* L.) leaf terminals, but a recent study (Sullivan and Foster, 2013) did not detect chicoric acid in rhizoma peanut (*Arachis glabrata* Benth.) leaves, though different species and fractions were examined. Only two studies have found chicoric acid found in fruit (Chinese firethorn, Zhao et al., 2013; zucchini, Iswaldi et al., 2013). Potential sources of chicoric acid for consumer products need to be validated and tools for quality monitoring for chicoric acid in crops and product manufacturing need to be developed.

## Plant × growing environment interactions

Plant responses that cause chicoric acid accumulation can vary among plant, plant fraction, and growing conditions. To date, there have not been comprehensive enough body of research in this area to draw broad scale inferences. Since the quality of any agricultural product begins in the field, applying the knowledge gained from research in this area is vital for product improvements and production efficiency for growers and processors alike. A general review of managing phenolics in crop production is available (Treutter, 2010). This section below summarizes research how plant growing environment may specifically alter chicoric acid accumulation.

### Plant development and growing season

Plant development is known to alter accumulation of phenolic compounds. Some research has indicated that more mature tissue contains less chicoric acid than younger tissue. For example young, actively growing Neptune grass leaves contained more chicoric acid when compared to older leaves (Cariello and Zanetti, 1979; Agostini et al., 1998; Haznedaroglu and Zeybek, 2007).

Maximum accumulation of chicoric acid may also occur at a specific time in plant development. For example, *meso*-chicoric acid accumulation reversed in *E. arvense* during development of sporophytes, with a continual buildup until to peak concentration at ~130 days of growth after which followed a steady decrease to 220 days of growth, demonstrating that sporophyte development altered phenolic acid reserves (Hohlfeld et al., 1996). The effects of tissue age may also vary with plant taxon. For example, in sowthistle (*Sonchus olearaceus* L.), mature leaves contained higher levels of chicoric acid compared to close-to-the-base and young leaves (Ou et al., 2012).

For some plants, however, no consistent relationship was found between tissue age and chicoric acid production. For example, no consistent trend was seen in chicoric acid levels of outer loose portions of leaf lettuce, as they fluctuated during seven sampling periods, although no statistics were conducted (Romani et al., 2002). In contrast, phenolic acids detected by HPLC in 5 varieties of lettuce and one variety of endive cultivated in hydroponic solutions chicoric acid concentrations in changed substantially with time of year and plant age (Amimoto and Fukui, 1996).

Time of year can alter accumulation of phenolics beyond its effects on plant development. For example, *E. purpurea* and *E. pallida* roots contained higher levels of chicoric acid in late spring than in other development stages/season (early winter, early spring, summer, and mid autumn; Thomsen et al., 2012). An earlier study found that summer harvested *E. purpurea* samples had more chicoric acid than those harvested in the fall (Perry et al., 2001). In some studies, however, no consistent relationship was found between time of year and chicoric acid production. For example, different harvest times (winter vs. spring) did not alter chicoric acid among six lettuce cultivars that were grown in the greenhouse (Nicolle et al., 2004).

Separating out the effects of time of year and plant development on chicoric acid accumulation has been indirectly investigated. Red oak leaf lettuces were grown at different temperatures to evaluate the influence of cool cultivation on plant phenolic composition (Becker et al., 2013). Plants were grown in either at 10/15°C day/night (warm treatment) or 12/7°C day/night (cool treatments) for up to 52 days. Heads from cool-cultivated plants container higher concentration so chicoric acid than warm-cultivated plants 26 days after planting; however, this was interpreted a developmental difference between plants, not the direct effect of temperature on chicoric acid production.

Much of the research on chicoric acid production has used lettuce and *Echinacea* species or cultivars. Lettuce is an annual plant while *Echinacea* is a herbaceous perennial plant. Seasonal changes in composition in annual and perennial plants can differ substantially and their metabolism can be regulated by different intrinsic (genetic) and environmental triggers. Many of the seemingly conflicting reports concerning effects of tissue maturity and growing season on chicoric acid accumulation may be related to whether the plants being evaluated are annual or perennial in nature. Accumulation of phenolic acids in plants are the net end product of their anabolism and catabolism. The reason for anabolism and catabolism of chicoric acid during plant growth is unknown. For growers and producers, these fluctuations in chicoric acid levels with tissue maturation and throughout the growing season should be taken into consideration before scheduling harvest dates or planning herbal product manufacturing (Perry et al., 2001).

### Cultivation

Domestication of crops, through its effects on growing environment and breeding/selection can alter plant composition. Agronomic production conditions may decrease chicoric acid production compared to plants growing in their natural environment. Chicoric acid disappeared in some *Echinacea* species after wild-collected plants were transplanted in a greenhouse (Binns et al., 2002). For example, *E. laevigata* roots accumulated chicoric acid after transplantation, but the opposite trend was observed in *E. pallida*, *E. paradoxa*, and *Echinacea* hybrids. In *E. sanguinea* Nutt., chicoric acid levels decreased in the roots as flower-head chicoric acid level increased during flower maturity. Wild *Echinacea* flower heads had more overall chicoric acid than cultivated heads (Binns et al., 2002).

Decreased accumulation of chicoric acid between wild plants and those grown in production systems may be simply related to greater stresses in the wild increasing phenolic accumulation (Dixon and Paiva, 1995). Similarly, environmental differences among production systems may alter plant stress responses, including production of chicoric acid. For example, chicoric acid concentrations in field grown lettuce were >2 times greater than plants grown in a polycarbonate greenhouse 16 days after planting (Romani et al., 2002). Other environmental differences between agronomic systems and the natural environment that are not directly related to plant stress may also play a role. For example, higher chicoric acid levels occurred with increased elevation in a small number (*n* = 7) of smooth hawksbeard (*Crepis capillaris* L. Wallr.) flower heads collected from altitudes of 180–1060 m (Zidorn et al., 2005). Although this trend was not observed in fall dandelion flower head samples (*n* = 24) that had been collected at elevations from 10 to 2480 m (Grass et al., 2006).

Many proponents of organic agriculture liken it to more natural growing conditions for plants. Organic production systems have been reported to have no effect or increase chicoric acid in certain crops. “Verde” zucchini (*Cucurbita pepo* L.) samples from a public marketplace contained chicoric acid only when fruit were identified as organically grown (Iswaldi et al., 2013). In contrast, growing green leaf lettuce plants grown in conventionally managed field plots had similar chicoric acid concentrations and dry weight as plants grown in 5 year old certified organic plots when plants were fertilized (Rajashekar et al., 2012). Interestingly, in the same study, growing plants with organic fertilizer (composted cattle manure and alfalfa hay) decreased chicoric acid concentrations compared to plants grown without additional fertilizer and, plants grown with non-organic fertilizer in non-organic plots had similar chicoric acid concentrations and greater dry weight than plants grown without additional fertilizer. These results suggest the differences in chicoric acid accumulation reported maybe a function of nutritional stress. Research on industrial bioproduction of chicoric acid indirectly lends support to increased stress increasing chicoric acid accumulation. Chicoric acid production by adventitious roots of *E. purpurea* cultured for 50 days in bioreactors was almost 4 times greater than concentrations in roots from field-grown plants (Wu et al., 2007b). Adventitious root production is considered a stress related response in many plants and causes an up-regulation of phenolic metabolism. There is an obvious need for more research on how chicoric acid accumulation in plants is regulated and how cultivation and production strategies can be used to optimize accumulation in plants.

### Microorganisms

Interactions between plants and microorganisms can cause an astounding variety of effects on plant composition that may result in accumulation of phenolic compounds due to stress or altered plant vigor. Pathogens can have a negative impact on plant growth but may increase phenolic accumulation. For example pathogen attack (cucumber mosaic virus and phytoplasma-prokaryote) decreased chicoric acid levels in *E. purpurea* roots (Pellati et al., 2011). Foliar application of carboymethyl chitin glucan (a fungal elicitor produced by *Penicillium*) increased production of chicoric acid in roots of *E. purpurea* (Hudec et al., 2007). Increased chicoric acid accumulation in response to pathogen infection or pathogen elicitors supports a link between plant stresses and enhanced chicoric acid production. The linkage between pathogen infection and chicoric acid production has received little research attention with plants.

Beneficial bacteria and fungi (including mycorrhizal fungi) can enhance plant growth but have been shown to have positive, negative, and no influence on accumulation on plant phenolic composition. For example, arbuscular mycorrhizal fungus (AMF) colonization did not alter chicoric acid levels in either “Genovese Italian” or “Purple Petra” basil leaves and stems (Lee and Scagel, 2009). In contrast the shoots of *E. purpurea* showed no change in chicoric acid levels due to AMF colonization, but the roots had more chicoric acid with colonization (Araim et al., 2009). Increased chicoric acid accumulation in roots of AMF colonized plants was hypothesized to be a result of the plants reaction to fungal infection. In contrast, the effects of AMF on chicoric acid production in “Cinnamon,” “Siam Queen,” “Sweet Dani,” and “Red Rubin” basils, revealed that both AMF and phosphorus (P) fertilizer rate increased chicoric acid accumulation in basil shoots (Scagel and Lee, 2012). These results suggest that neither nutritional stress or the plant AMF infection consistently increases chicoric acid production. Inoculation of crop plants with mycorrhizal fungi is becoming a more routine practice in production systems for several crops. Improved knowledge of how these fungi may alter secondary plant metabolism is needed to understand whether these fungi can be used to manipulate target compounds such as chicoric acid.

### Light, temperature, and other stress elicitors

The direct effects of selected environmental stresses (stress elicitors like heat, light, ultrasound, hormone, etc.) on chicoric acid accumulation have been investigated experimentally in very few crops. In most, but not all cases, increased levels of treatments that resulted in greater plant stress increased chicoric acid production.

Five-week old “Baronet” lettuce plants were subjected to brief simulated environmental stresses and sampled 1 day before and <1 day, 1 day, and 3 days after stress (Oh et al., 2009). A brief heat shock (40°C for 10 min at 90% relative humidity, RH) increased chicoric acid 3 days after treatment compared to control plants. Cold treatment (4°C for 1 day in a growth chamber) increased chicoric acid 1 h, 1 day, and 3 days after treatment. High light (800 μmol m^−2^ s^−1^ for 1 day) exposure had no influence on chicoric acid 1 h after exposure, but increased concentrations 1 day and 3 days after exposure. On average the heat and cold stress treatments resulted in ~2 fold increase in chicoric acid while high light exposure increased concentrations by ~7 fold.

Reducing photosynthetic photon flux density (PPFD) from 410 μmol to 225 μmol m^−2^ s^−1^ had no influence on chicoric acid concentrations in red oak leaf lettuce (“Eventai”) even though lower light decrease plant dry weight and accumulation of reducing sugars in 4 week old plants (Becker et al., 2013).

Manipulation of the growing environment in bioproduction of chicoric acid has been investigated. Manipulating growth temperatures (10, 15, 25, 20, and 30°C) and photoperiods (24 h light/day to 24 h dark/day; 40 μmol m^−2^ s^−1^ fluorescent light) in *E. purpurea* suspension cell cultures revealed that 20°C and 3 h light and 21 h dark for 5 weeks produced the most chicoric acid (Wu et al., 2007a). Chicoric acid still accumulated in *E. purpurea* cultures that were grown in complete darkness for 5 weeks. Unfortunately no statistics were performed on the results. Ultrasound treatments successfully were used to enhanced chicoric acid production in hairy root cultures of *E. purpurea* (Liu et al., 2012). In field and controlled environment production systems, use of known elicitors of plant stress metabolism have been evaluated for their effects on chicoric acid production. Foliar application of stress or defense elicitors (acetyl salicylic acid, ASA; salicylic acid, SA; and methylsalicylic acid, MSA) applied at 0, 10, 100, and 1000 μ M concentrations to *Rauvolfia serpentine* increased chicoric acid production in shoots and roots of plants grown in a controlled environment conditions (Nair et al., 2013). In this study, the most dilute elicitor solutions had the greatest influence on chicoric acid in shoots while more concentrated elicitor solutions had the greatest effect on chicoric acid in roots. Of the three elicitors evaluated SA had the greatest influence on chicoric acid. In contrast, other phenolics (e.g., chlorogenic acid, caftaric acid) were more sensitive to MSA. A similar study evaluated the effects of foliar application of ASA, SA, MSA, and titanium (IV) ascorbate at different concentrations on chicoric acid in *E. purpurea* growing in the field for 2 years (Kuzel et al., 2009). In comparison to controls, chicoric acid concentrations in shoots were only greater in plants treated with SA at 10 μ M and to a lesser extent titanium (IV) ascorbate. All elicitors increased chicoric acid in roots compared to controls.

### Salinity and nutrients

Nutrients and salinity are intimately linked in production of crops in field and controlled culture. A relatively large body of literature is available on the effects of plant nutrition on production and regulation of other several phenolic compounds see review by Treutter (2010). However, the direct effects of specific salinity rates (as assessed by electrical conductivity, EC) and selected nutrients on chicoric acid accumulation have not been investigated experimentally in many crops, and reported results vary. Some studies assessing how salinity and nutrients influence chicoric acid only report concentrations and not total content; therefore in these studies it is impossible to determine whether treatments altered production or the differences in chicoric acid are only a result of treatments on plant growth.

Sensitivity of chicoric acid production to salinity varies among species, with evaluated levels of salinity having negative, positive, and no effect of chicoric acid accumulation. Two species of *Echinacea* (*E. purpurea* and *E. pallida*) expressed chicoric acid concentrations differently in response to salinity (0, 50, 75, and 100 μm sodium chloride hydroponic solution) (Sabra et al., 2012). Chicoric acid concentrations in *E. purpurea* increased considerably with salt concentration up to 75 μm, but was lowest at the greatest (100 μm) salinity; while in *E. pallida* chicoric acid concentrations were elevated in both of the higher (75 and 100 μm) salinity treatments (Sabra et al., 2012). Salinity treatments (NaCl, 5 and 50 mol m^−3^) decreased plant dry weight and chicoric acid in leaves but increased chicoric acid in roots of 4-month-old *E. angustifolia* grown in the field (Montanari et al., 2008).

In contrast to the results in the *Echinacea* study above, baby romaine lettuce showed no differences in chicoric acid concentration after growth in various salinities (2.8, 3.8, and 4.8 dS m^−1^) and storage (4°C for 10 days; Chisari et al., 2010). In another study with hydroponically grown “Capitata” lettuce increasing salinity from 0 to 150 mM NaCl increased concentrations of total phenolics and the individual phenolic acids identified (including chicoric acid) after 10 days of salinity treatment (Garrido et al., 2014). Increased salinity increased concentration of phenolic acids by 22.4% but no data was presented for the individual phenolic acids; additionally, increased salinity decreased plant biomass therefore the salinity may not have altered the production of chicoric acid. In a study with 5 varieties of lettuce and one variety of endive cultivated in hydroponic solutions with 2.4 and 4.8 mS cm^−1^, phenolic acids detected by HPLC in indicated that there were no qualitative differences in phenolic acids at different EC; however, chicoric acid concentrations in lettuce was decreased at the highest EC (Amimoto and Fukui, 1996).

Sensitivity of chicoric acid production to various forms of fertilizer varies among species, with evaluated fertilizer forms having negative, positive, and no effect of chicoric acid accumulation. In some research nutrient application rate was also altered with fertilizer form; therefore the effects of fertilizer form on chicoric acid cannot be separated from the differences in nutrient application rates among treatments. Fertilizer additions (organic or non-organic) decreased chicoric acid accumulation in green leaf lettuce (“Baronet”) (Rajashekar et al., 2012). Use of a commercial form of cow manure vs. conventional fertilization did not result in chicoric acid differences within chicory leaves, in either water stressed plants or unstressed ones (Heimler et al., 2009). Nitrogen (N) source in fertilizer (nitrate vs. mix of ammonium and nitrate) had no influence on growth of 4-month old *E. angustifolia*, but plants grown with only nitrate had higher concentrations of chicoric acid in leaves and roots than plants grown with a mix of nitrate and ammonium (Montanari et al., 2008). Growing *E. pallida* and *E. purpurea* at 3 different fertilizer rates for 7 months in a field planting had no significant influence of fertilizer rate on root weight or chicoric acid concentration although root growth and chicoric acid concentration of roots tended to decrease with increasing fertility (Dufault et al., 2003).

Increased P rate enhanced chicoric acid production in basil and shoot accumulation of chicoric acid was correlated with enhanced uptake of P, Ca, Mg, B, Fe, and Mn (Scagel and Lee, 2012). Chicoric acid production in basil did not correlate with the uptake of K or Zn even though production of other rosmarinic acid (the main phenolic in basil) correlated with uptake of these nutrients (Scagel and Lee, 2012). In contrast, K treatment (0.005 M) increased chicoric acid in basil leaves after 30 days of post-germination growth (Nguyen et al., 2010).

## Post-harvest handling, processing, and storage

Optimizing processing steps to better extract or retain chicoric acid directly improves commercial product quality (Lee, 2010). Chicoric acid has been proposed as a quality control indicator compound due to its rapid degradation, in contrast to other secondary metabolites, within plant materials (Stuart and Wills, 2003; Lee, 2010). This section summarizes how different post-harvest and processing conditions influence chicoric acid retention.

### Post-harvest reaction of plant tissues

Harvesting procedures that purposefully wound the plant can elicit stress responses from plants that may enhance accumulation of phenolic compounds, including chicoric acid. Chicoric acid levels of iceburg lettuce midrib increased from harvest until day 2 of storage at 7°C (Luna et al., 2012). Iceburg lettuce butt (cut stem/midrib end after harvesting) started to produce chicoric acid 48 h after the initial wounding, and showed biosynthesis highest at the wound site, then progressively decreasing up the stem away from the wound site, to eventually being undetectable (Tomas-Barberan et al., 1997). They also showed that applying a calcium solution (0.30 M calcium chloride) to the wound site inhibited browning (visually undesirable trait) on the stem surface while retaining chicoric acid. These results suggest that chicoric acid production is linked to wound response.

In contrast wounding the white, green, and red tissues of “Lollo Rosso” lettuce increased chlorogenic and caffeoyltartaric acids but had no influence on chicoric acid (Ferreres et al., 1997). Differences in wound-induced phenolic responses among lettuce varieties or tissues may be a function of the pre-existing abundance of phenolics prior to wounding and different levels of native enzymes. Tissues or varieties with an abundance of phenolics were hypothesized to have a lower or less detectable wound-induced response in phenolics.

### Washing and rinsing

Post-harvesting procedures used to clean material and decrease potential contamination as part of the processing chain (washing and rinsing) can alter the phenolic composition of plant tissues. Of the few studies done in this area of research, mostly on lettuce, washing or rinsing plant material after harvest has little effect on chicoric acid retention. There was no treatment differences in chicoric acid levels in rinsed (control, ozonated water, ozonated with UV light treatment, and chlorinated water) shredded iceburg lettuce after air storage (Beltran et al., 2005). There were minimal changes (or differences) in chicoric acid among iceburg lettuce samples after a washing regime (a number of different preparations; cut then rinsed, rinsed then cut, chlorine free tap water, 100 ppm chlorinated water, or ozonated water, etc.) (Baur et al., 2004). Shredded lettuce chicoric acid levels did not alter between washing treatments (tap water rinsed, distilled water dipped, and 100 ppm chlorinated water dipped), but as this study made no measurement of pre-shredded lettuce, loss or retention cannot be determined (Kenny and O'Beirne, 2009).

### Storage

Post-harvesting storage, prolong availability of plant tissues over time, can alter the phenolic composition of plants tissues. For lettuce, storage conditions for plant material after harvest decreases, increases, or has little effect on chicoric acid retention. Concentrations of chicoric acid in shredded leaves of iceberg lettuce decreased after modified atmosphere storage (mixture of oxygen and carbon dioxide) of 13 days at 4°C (Beltran et al., 2005). There were minimal changes (or differences) in chicoric acid among iceburg lettuce samples after 9 days of dark storage at 4°C (Baur et al., 2004). Five of the six lettuce cultivars evaluated after 7 days of 5°C storage increased in chicoric acid content (Cantos et al., 2001), indicating chicoric acid can be biosynthesized in lettuce tissue during post-harvest storage. After 24 h storage at 4°C, chicoric acid in fresh cut leaves of lettuce purchased from local marketplaces increased (~2 fold), then decreased to the original level after 72 h storage (Degl'Innoocenti et al., 2008). In contrast, chicoric acid concentration increased in fresh cut endive leaves after 24 h storage and remained elevated by 72 h.

Modified atmosphere packaging (MAP; air replaced with 3% oxygen, 8% carbon dioxide, and 89% nitrogen) did not enhance chicoric acid retention from that of air in the red and green parts of the lettuce, though white lettuce tissues increased in chicoric acid with the MAP (Gil et al., 1998). MAP of fresh-cut romaine lettuce stored for 10 days reportedly increased chicoric acid in leaves and light exposure of package product had no influence on chicoric acid; however, only total phenolic content was presented (Martínez-Sánchez et al., 2011).

Similar disparate results have been reported for the effects of storage on *E. purpurea*. Lowered moisture/humidity during *E. purpurea* post-harvest storage increased chicoric acid retention of final products (Kim et al., 2000; Wills and Stuart, 2000). Storage of freeze dried *E. purpurea* at 10 and 20°C in polyethylene terephthalate/aluminum boil/polyethylene bags had no influence on chicoric acid during 180 days (Lin et al., 2011). Storage at 30°C decreased chicoric acid after 90 days. In the same study, storage of freeze dried *E. purpurea* at 40 and 60% RH in the dark did not influence chicoric acid during 180 days. Storage at 80% RH decreased chicoric acid after 135 days. Additionally, dark storage of freeze dried *E. purpurea* at 40% RH in nylon/polyethylene bags did not influence chicoric acid during 180 days. Light decreased chicoric acid production after 90 days.

### Drying

Drying is a commonly used food preservation method. Drying is used to preserve phenolics and other compounds (essential oils, etc.) in plant materials; however, the effects of drying will vary depending on the drying procedure, the type of plant material, and chemical in question (Lee and Scagel, 2009; Lee, 2010).

Freeze drying, though gentler than ordinary air drying, decreased phenolics in basil preparations by 13% compared to fresh material (Lee, 2010). Freeze drying of *E. purpurea* flowers retained chicoric acid better than vacuum drying, microwave drying, or air drying (Kim et al., 2000). Freeze dried flower heads also retained more color and had less browning (Kim et al., 2000). Drying *E. purpurea* for different lengths of time using vacuum freeze drying, cool air drying, and hot air drying revealed that vacuum freeze drying generally retained higher concentrations of chicoric acid and cool air drying greater than hot air drying (Lin et al., 2011). Others have demonstrated that microwave drying of *E. purpurea* retained more chicoric acid than steam heating, hot water bath, or air-drying (in decreasing order of chicoric acid retention; Zhang et al., 2011). Drying temperature can also play a role in retention of phenolics in plant materials. Higher air-drying temperature (70°C compared to 40 and 25°C) accelerated the loss of chicoric acid in *E. purpurea* flowers (Kim et al., 2000).

*Echinacea purpurea* aerial portions had a greater loss of chicoric acid than did roots during drying (Stuart and Wills, 2003). Different drying temperatures did not degrade *E. purpurea* alkamide in a manner like chicoric acid (Stuart and Wills, 2003), indicating the importance of monitoring all target compounds until their traits are fully understood. Over-drying had no effect on chicoric acid retention (Stuart and Wills, 2003).

### Additional minimal processing and additives

There are many other processing chain procedures that have the potential to influence phenolic composition of plant materials or products because of their use of heat, additives, preservatives, etc. that may alter phenolic stability. There have been few studies done in this area of research, and most studies, to date, have used *E. purpurea*. High hydrostatic pressure (HHP) pasteurization to increase product microbial stability did not alter chicoric acid retention in *E. purpurea* flowers and roots over to unpasteurized samples (Chen et al., 2010). Additives like citric acid, malic acid, and dried Hibiscus (*Hibiscus sabdariffa* L.) flower glycerin extract increased the retention of chicoric acid (and other bioactives) in *E. purpurea* extracts (in glycerin) for 4 months at 25°C (Bergeron et al., 2002). Additives, including 0.05 M ascorbic acid, 0.10 M ascorbic acid, 30% ethanol, and 40% ethanol, helped *E. purpurea* extracts to maintain a consistent chicoric acid concentration for 4 weeks (Nüsslein et al., 2000).

### Cooking

The consumer method for use of plants and plant products that may have the greatest potential to alter their phenolic composition is cooking. Cooking can used by the consumer on fresh materials as prior to consumption or storage. Boiling plant materials prior to consumption can increase, decrease, or have little effect on chicoric acid in chicory. Little chicoric acid was lost after boiling chicory leaves for 30 min. (Innocenti et al., 2005). Boiling stems for 8 min decreased the concentration of total phenolics in stems of “Galatina” chicory, but had no effect on concentration of total phenolics in stems of “Molfettese” chicory (Renna et al., 2014). In contrast, microwaving stems of both chicory varieties in water for 3 min increased concentration of total phenolics in stems (Renna et al., 2014). These authors hypothesized that these changes in total phenolics may reflect how the cooking techniques evaluated effected chicoric acid, the primary phenolic constituent of total phenolics in chicory (Innocenti et al., 2005).

Blanching harvested basil prior to freezing preserved chicoric acid levels better than basil frozen without an initial blanching step (Lee, 2010). Four *Ocimum* species total phenolics were compared after five cooking methods (blanching, boiling, steaming, sautéing, and high temperature via pressure-cooking) to control-fresh, though chicoric acid was not separately identified (Trakoontivakorn et al., 2012). HPLC profiles showed that pressure-cooked basil had the greatest loss of phenolics (Trakoontivakorn et al., 2012).

Additional work on chicoric acid levels remaining in the edible portions after cooking is needed. The lack of literature in this area is not surprising since most of the edible plant parts listed in Table 1 are typically eaten raw or minimally processed (ready-to-eat salad mixes), as a tea, as pre-prepared herbal liquid extracts (alcohol free, contains alcohol, etc.), or as capsules.

## Biological activities of chicoric acid

Chicoric acid properties have been reported to include anti-cancer, anti-obesity, antiviral, and anti-diabetic (King and Robinson, 1998; Pluymers et al., 2000; Charvat et al., 2006; Queffelec et al., 2008; Tousch et al., 2008; Tsai et al., 2012; Azay-Milhau et al., 2013; Xiao et al., 2013). For example, chicoric acid and its analogs have been claimed to possess anti-Human Immunodeficiency Virus (HIV) activity due to its involvement in HIV integrase inhibition, which could perhaps hinder HIV strain replication (King and Robinson, 1998; Charvat et al., 2006; Queffelec et al., 2008). Bel-Rhlid et al. (2012) reported a means of chicoric acid hydrolysis, facilitated by the probiotic bacterium (*Lactobacillus johnsonii*) after ingestion, but prior to absorption and metabolism. Due to the concerns surrounding all *in vitro* antioxidant measurements, chicoric acid antioxidant studies will not be summarized here, but the issues and research needed in this area have been well summarized elsewhere (Verhagen et al., 2010; Chiva-Blanch and Visioli, 2012).

Care should be taken when examining biological activities of a whole plant, due to the many bioactive components it likely has. For example, in *E. purpurea*, chicoric acid is only one of the several compounds (alkamides-also known as alkylamides, other caffeic acid derivatives, polysaccharides, and glycoprotein) associated with purported health benefits of *E. purpurea* supplements (Barnes et al., 2005). But, a specific genus and species reported to contain a high concentration of chicoric acid does not guarantee that other bioactive compounds are high in the plant as well. For example, chicoric acid was found highest in wild grown old inflorescences of *E. sanguinea*, while alkamides were highest in roots of greenhouse cultivated *E. purpurea* originally collected from the wild (Binns et al., 2002). It should be noted that the immunostimulating properties of *Echinacea* products are still under investigation, and the evidence to date indicate its bioactivities were no better than placebo (Barnes et al., 2005; Gertsch et al., 2011; and references there in).

Consumer education and awareness in healthy eating has resulted in a surge in dietary phenolic consumption and market demand for these products. Although this review is only focused on the phenolic compound- chicoric acid, any health benefits of plants mentioned in this review require more data. For those wishing to supplement chicoric acid intake, it is still unclear which delivery form (food, pill, capsules, extract, etc.), or what level would be most effective for improving human health that minimizes possible risks of toxicity, herb/drug interactions, or synergistic and/or antagonistic effects. Despite excitement about chicoric acid's potential from antiviral researchers, or individuals and families affected by HIV, additional research is needed to understand the benefits of chicoric acid and its analogs.

## Concluding remarks

There is a strong need for a well-defined dietary supplement regulation in the US. Until dietary supplements receive the scrutiny as foods in the US, with minimum quantities of active ingredients, full ingredient disclosures, safety guidelines, sound science supporting claims, etc. the safest source of consuming chicoric acid is via food. Good review articles (Dufault et al., 2000; Cardellina II, 2002; Sanzini et al., 2011; Khan and Smille, 2012; Applequist and Miller, 2013) regarding these concerns and possible ways to improve our current US dietary supplement situation are available. Effective research and commercial use of plant derived chemicals requires well defined and validated chemical analyses (Lee, 2014). Methods for chicoric acid detection and quantification have improved substantially over the last 25 years; however, future work is needed to clarify the biosynthesis pathway of chicoric acid and the role of chicoric acid in the plant. Additionally, potential consumer benefits from chicoric acid can only be effectively realized after we have improved knowledge of growing and processing conditions for maximum retention, and how effects of domestic cooking may alter compound activity. As a supplement, there is a great deal of room for improvement in our knowledge of chicoric acid.

### Conflict of interest statement

The authors declare that the research was conducted in the absence of any commercial or financial relationships that could be construed as a potential conflict of interest.
